# Sonosensitive Phase-Changeable Nanoparticle Mediated Enhanced Chemotherapy in Prostate Cancer by Low-Intensity Focused Ultrasound

**DOI:** 10.3390/ijms24010825

**Published:** 2023-01-03

**Authors:** Junyong Dai, Yunfang Wu, Ziqun Chen, Linkang Xiao, Weili Zhang, Yang Cao

**Affiliations:** 1 Chongqing Key Laboratory of Ultrasound Molecular Imaging, Institute of Ultrasound Imaging, Department of Urology Surgery, Second Affiliated Hospital, Chongqing Medical University, Chongqing 400010, China; 2 Chongqing University Cancer Hospital, Chongqing 400044, China; 3 Chongqing Wanzhou District Maternal and Child Health Hospital, Chongqing 404197, China; 4 Chongqing General Hospital, Chongqing 400013, China

**Keywords:** prostate cancer, phase-changeable nanoparticles, chemotherapy, low-intensity focused ultrasound

## Abstract

Prostate cancer (PCa) is one of the most common cancer types. Early detection of PC offers the best chance of successful treatment. A noninvasive, image-guided therapy mediated by targeted nanoparticles (NPs) has the potential to improve the efficacy and safety of cancer therapies. Herein, we report a sonosensitive nanoparticle modified with anti-PSMA (prostate-specific membrane antigen) antibodies to activate target prostate tumors. These nanoparticles (PFP@IR780@PTX@liposome NPs) were co-loaded with the chemotherapeutic agent docetaxel and the sonosensitizer IR780, as well as phase-changeable perfluorocarbon (PFC) liquids. The liquid–gas phase change could be induced by low-intensity focused ultrasound (LIFU) in vitro. We found that the PFP@IR780@PTX@liposome NPs can specifically accumulate in prostate tumors after LIFU irradiation, as monitored by ultrasound and photoacoustic imaging. Meanwhile, docetaxel was controllably released from the nanoparticles to achieve enhanced chemotherapeutic therapy in vivo. These sonosensitive phase-changeable NPs can visually treat prostate cancers effectively and have a clinical potential.

## 1. Introduction

Prostate cancer (PCa) is the second most common cancer affecting men [1]. The incidence of prostate cancer has risen sharply in the last two decades. Thanks to advanced early diagnosis technology, the five-year survival rate of PCa patients has reached 98.9% [1]. Therefore, early detection and treatment are of great importance for prostate cancer [2]. Currently, all diagnostic and therapeutic approaches for prostate cancer still rely on highly invasive and nonspecific techniques, including random biopsies, irradiation, and radical surgeries [3], and chemotherapy causes many toxic side effects afterward. Therefore, it is urgent to find a biosafety and noninvasive method to overcome the limitations of surgery, radiation, and nonspecific chemotherapy. The development of nanotechnology may provide unprecedented opportunities to solve the challenges in prostate cancer via image-guided therapy. Liposome nanoparticles with lipid-encapsulated fluorocarbon perfluorocarbon (PFC) as the core can pass through the blood vessels of prostate cancer, enter tumor tissues, and bind to tumor cells, especially aggressive prostate cancer cells, and produce liquid–gas phase transitions triggered by ultrasound irradiation. Owing to acoustic droplet vaporization (ADV), acoustical microbubbles are produced and accumulate around cancer cells for a long time, benefitting early diagnosis and treatment. Ultrasound (US) has the advantages of being minimally invasive and having deeper penetration. It can be used to control the release of drugs and promote the penetration of nanoparticles or drugs into the tumor center through the cavitation effect.

Prostate-specific membrane antigen (PSMA), a human prostate cancer marker, is a type II transmembrane protein that exists in the cell membrane of prostate epithelial cells. It has high tissue specificity and is highly expressed in most prostate cancers, especially castration-resistant prostate cancer cells. The intracellular and extracellular segments of PSMAs, which have multiple epitopes, are the most meaningful target proteins for the diagnosis and treatment of prostate cancers.

Docetaxel is one of the most commonly used drugs to treat prostate cancers in the clinic, and it is also the most widely used therapeutic agent approved by the Food and Drug Administration (FDA) [4,5]. However, it may produce significant adverse reactions, which require limiting the dose and treatment duration. High doses (80 mg for 7 days) would cause more than 50% of patients to suffer from dose-limiting or treatment-limiting side effects and the termination of the therapy [6]; even if the dose is low (60 mg for 5 days), patients also have moderate and severe toxicity [7]. Thus, image-guided nanocarrier systems for targeted drug delivery can effectively avoid the toxicity and side effects of drugs and improve the efficacy of prostate cancer chemotherapy. Liposomes have a bilayer structure, similar to phospholipid bilayers in cells, and are selective for drug loading and have been used clinically to treat diseases [8,9].

IR780 iodide, a hydrophobic heptamethine indocyanine dye, has recently been explored as a light- or ultrasound-responsive agent for cancer treatment [10,11]. It interacts effectively with near infrared (NIR; 750–1000 nm) light, ensures sufficient light penetration within the biological transparent window, and minimizes the interaction between light and biological components [12]. IR780 has been considered a versatile photo-responsive or sono-responsive molecule that can be used for cancer sonodynamic therapy (SDT), photodynamic therapy (PDT), photothermal therapy (PTT), and real-time imaging [13,14,15]. Moreover, IR-780 has been recently characterized to exhibit preferential accumulation in the mitochondria of tumor cells. 

Herein, we report ultrasound-responsive drug controlled release from PSMA-targeted PFP@IR780@PTX@liposomes conjugated to anti-PSMA antibody. Docetaxel and IR780 were both encapsulated in the phase-changeable liposomes. Using low-intensity focused ultrasound, the delivery of docetaxel to the prostate tumors and the antitumor effects of PFP@IR780@PTX@liposome were evaluated in vitro and in vivo. Overall, we aimed to evaluate the therapeutic effects of sonosensitive phase-changeable nanoparticles with PSMA selective recognition. This could be used for ultrasonic and photoacoustic imaging-guided chemotherapy of prostate cancers.

## 2. Results

### 2.1. Characterization of PSMA-Targeted PFP@IR780@PTX@liposomes

**Figure 1:**
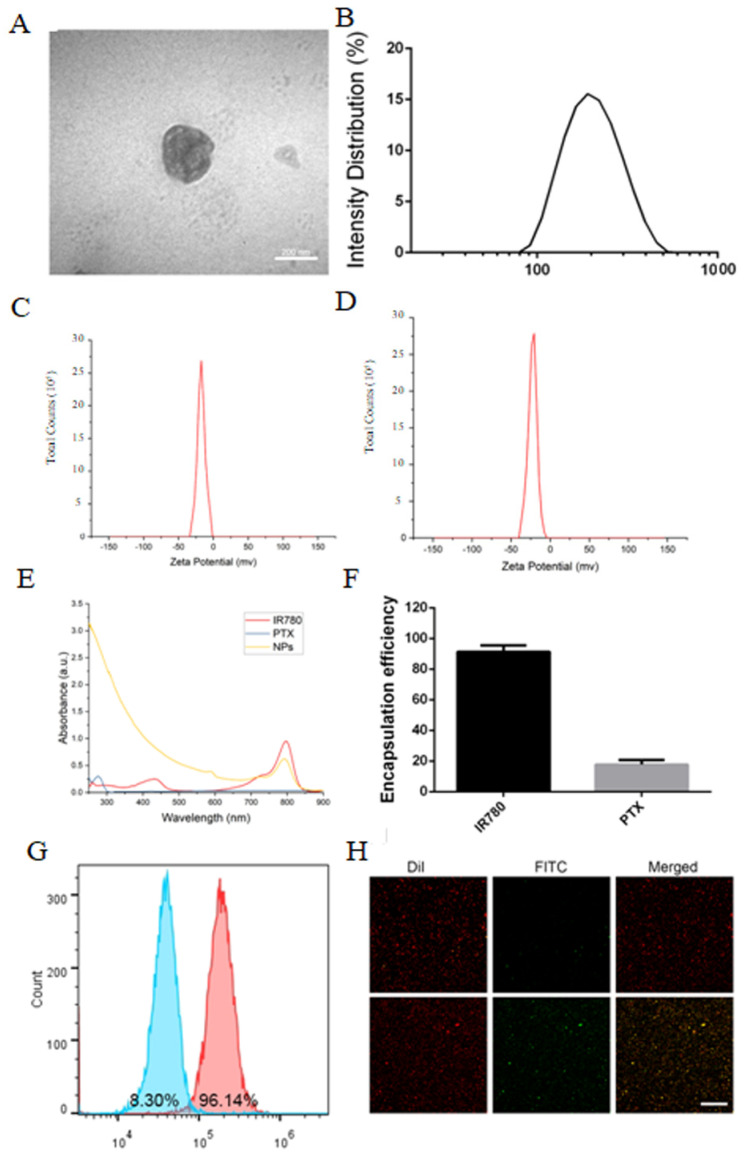
Characterization of PSMA−targeted PFP@IR780@PTX@liposomes: (**A**) TEM image; (**B**) Size distribution; (**C**) Zeta potential of the untargeted nanoparticles. (**D**) Zeta potential of the PSMA−targeted nanoparticles. (**E**) The absorption spectra of IR780, PTX, and PSMA−targeted nanoparticles. (**F**) The encapsulation efficiencies of IR780 and PTX. (**G**) The connection efficiency of PSMA and nanoparticles by flow cytometry (Left: control; Right: PSMA−targeted nanoparticles). (**H**) CLSM (Up: control; Down: PSMA−targeted nanoparticles). Scale bar: 50 μm.

PFP@IR780@PTX@liposomes showed a typical spherical structure under transmission electron microscope (TEM) (Figure 1A) and a diameter of approximately 226.4 ± 48.2 nm (Figure 1B). The surfaces of the nanoparticles were negatively charged (Figure 1C,D). The zeta potential changed from −16.9 mV to −24.7 mV after PSMA modification. Next, the loading of IR780 and docetaxel was examined using ultraviolet (UV) and high-performance liquid chromatography (HPLC). The results showed that the loading efficiency of PTX was 17.77% and that of IR780 was as high as 91.2% (Figure 1F). Then, the conjugation of nanoparticles and anti-PSMA antibodies was observed using flow cytometry and confocal laser scanning microscopy (CLSM). As shown in Figure 1G,H, the connection rate was 96.14%, with IR780-loaded NPs shown in red, FITC-labeled PSMA antibodies shown in green, and the merged images shown in yellow in CLSM. The results indicated that the nanoparticles and antibodies were connected successfully.

### 2.2. Phase-Transformation and Ultrasound Imaging of PSMA-Targeted PFP@IR780@PTX@liposomes In Vitro

**Figure 2:**
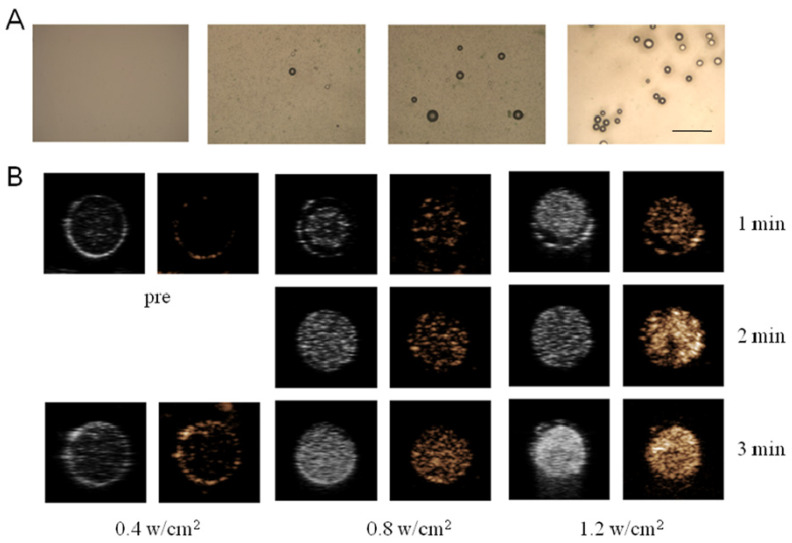
Phase transformation of nanoparticles triggered by LIFU: (**A**) Light microscope images of nanoparticles under various LIFU irradiation. (scale bar = 50 μm) (**B**) US images in B-mode and CEUS mode of nanoparticles (5.0 mg/mL) before and after LIFU irradiation.

The ultrasound-induced liquid–gas phase transition of PFP@IR780@PTX@liposomes was observed by microscopy (Figure 2A). Low-intensity focused ultrasound (LIFU) with low-intensity ultrasound energy could focus on specific parts and accurately trigger the liquid–gas phase transition on targets, preventing potential damage to surrounding tissues and minimizing adverse side effects. We used an ultrasound image analyzer (DFY-II type) to further evaluate the B-mode and contrast-enhanced ultrasound (CEUS) mode imaging of PFP@IR780@PTX@liposomes. The results indicated that the gray scale values of the B-mode and CEUS mode imaging were concentrated and acoustic intensity dependent. At the same, for the nanoparticle concentration, the gray value of the ultrasonic image with an intensity of 1.2 w/cm^2^ was significantly higher than that of 0.4 w/cm^2^. When the acoustic intensity was fixed, the CEUS signal increased with increasing nanoparticle concentration, indicating that PFP@IR780@PTX@liposomes can be a CEUS agent under LIFU irradiation.

### 2.3. Cytotoxicity Assay and Targeting of PFP@IR780@PTX@liposomes In Vitro

**Figure 3:**
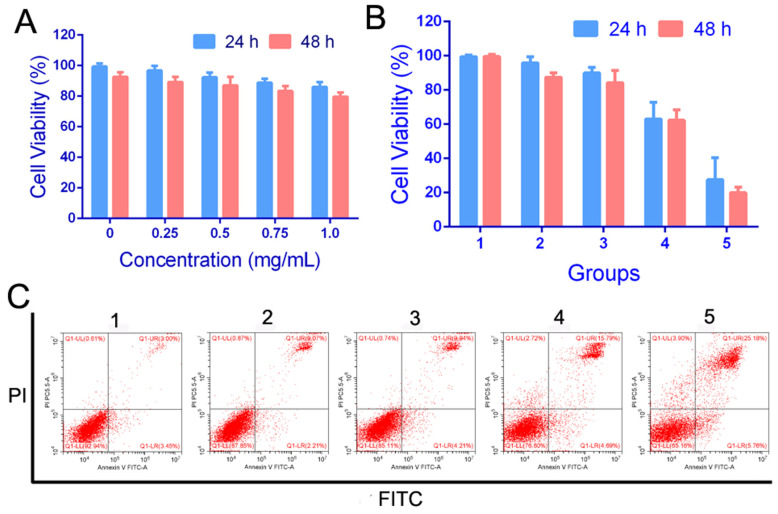
(**A**) Cytotoxicity of PFP@IR780@liposomes in vitro. (**B**,**C**) Cell apoptosis and FCM of C4-2 cells treated with different nanoparticles (1:PBS, 2: PTX, 3: PBS+LIFU, 4: PFP@IR780@PTX@liposomes, 5: PFP@IR780@PTX@liposomes +LIFU).

The CCK-8 method was used to evaluate the cytotoxicity of nanoparticles. As shown in Figure 3A, C4-2 cells were treated with various concentrations of PFP@IR780 @liposomes from 0 to 1 mg/mL for 24h or 48h. The results showed that, even if the concentration of nanoparticles reached 1 mg/mL, its cytotoxicity was very low, and the cell viability was more than 80%, suggesting that the fabricated nanoparticles had no obvious cytotoxicity to cancer cells. Then, the C4-2 cells were treated with different groups (PBS, PTX, PBS+LIFU, PFP@IR780@PTX@liposomes, and PFP@IR780@PTX@liposomes+LIFU). The CCK-8 assay and flow cytometry were utilized to detect cell apoptosis. The cell viabilities were the highest for the PFP@IR780@PTX@liposomes+LIFU group (Figure 3B). The results of the flow cytometry were consistent with those of the CCK8 assay. The apoptosis rate of tumor cells was the highest, 30.94%, in the PFP@IR780@PTX@liposomes+LIFU group (Figure 3C). The results indicated that PFP@IR780@PTX@liposomes had the best therapeutic effects under LIFU irradiation. Based on acoustic droplet vaporization (ADV) and ultrasound-targeted microbubble destruction (UTMD), the liquid–gas phase transition of the liquid fluorocarbon was activated using focused ultrasound, resulting in the controlled release of the encapsulated PTX (Figure S1). Additionally, it increases the permeability of the cell membrane and further enhances the killing effect of drugs on cells.

**Figure 4:**
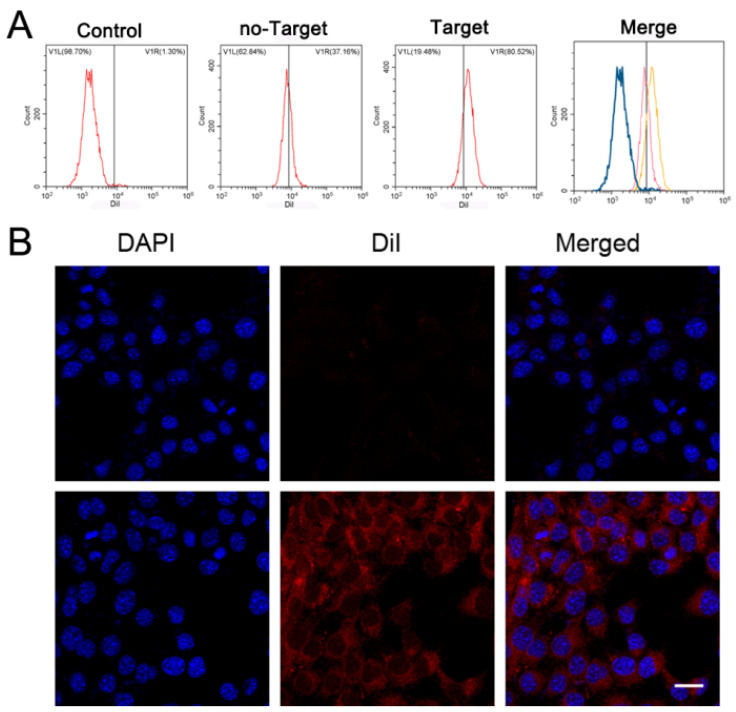
Cell-targeting experiments: (**A**) The connection between NPs and cells as detected by flow cytometry. (**B**) 2D images of the connection between NPs and cells observed by LSCM. Up: control; Down: PSMA-targeted nanoparticles. Scale bar: 20 μm.

The endocytosis of PSMA-targeted nanoparticles was evaluated using flow cytometry (Figure 4A). The endocytosis efficiency of PSMA-targeted PFP@IR780@PTX@liposomes was significantly higher than that of the untargeted PFP@IR780@PTX@liposomes group (80.52% vs. 37.16%). CLSM also showed the same trend (Figure 4B); the PSMA-targeted groups presented as bright red light in cells, while the light was weak for untargeted nanoparticles, indicating that nanoparticles could aggregate into prostate tumor cells actively and efficiently through PSMA targeting modification to achieve their functions.

### 2.4. Ultrasound and PA Imaging In Vivo

**Figure 5:**
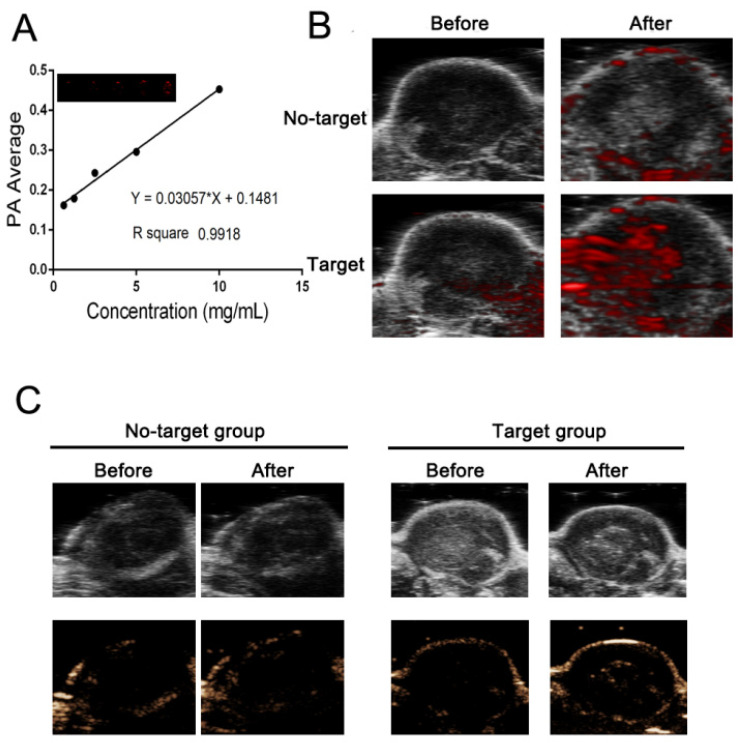
PA and ultrasound imaging in vivo: (**A**) Quantitative analysis of the relationship between the PA signal intensity and the nanoparticle concentration. (**B**) PA images of the tumor before and after nanoparticle injection. (**C**) US images in B-mode and CEUS mode of nanoparticles before and after LIFU irradiation.

Due to the light absorption ability of IR780, we performed photoacoustic imaging of PFP@IR780@PTX@liposomes at different concentrations, as shown in Figure 5A. The PA signal was dose-dependent with an excellent linear relationship (R^2^ = 0.9918). The in vivo tumor imaging was also evaluated. PSMA-targeted PFP@IR780@PTX@liposomes were injected into tumor-bearing mice via the tail vein, and untargeted PFP@IR780@PTX@liposomes were used as the control (Figures S2 and S3). Thanks to PSMA-targeted modification, targeted nanoparticles can specifically aggregate in tumor regions compared with the control group (Figure 5B). According to the ultrasound imaging ofPFP@IR780@PTX@liposomes in vitro, the in vivo ultrasound imaging and diagnostic potential of phase-changeable nanoparticles were studied. As shown in Figure 5C, PSMA-targeted nanoparticles could accumulate effectively in tumor regions and had a great CEUS imaging ability. 

### 2.5. In Vivo Antitumor Efficacy

**Figure 6:**
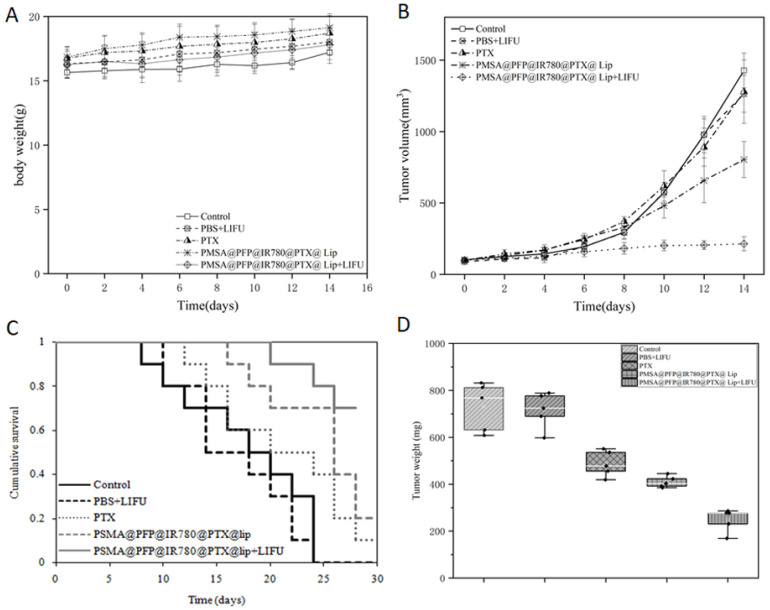
Antitumor efficacy of NPs in vivo: (**A**) body weight, (**B**) tumor volume, (**C**) cumulative survival of animals, and (**D**) tumor weight of nude mice subjected to different treatments. Error bars represent the means ± SD for n = 3.

To investigate the antitumor effect of PSMA-targeted PFP@IR780@PTX@liposomes in vivo, xenograft-bearing nude mice were treated with saline, saline plus LIFU irradiation, PTX, PFP@IR780@PTX@liposomes, and PFP@IR780@PTX@liposomes plus LIFU irradiation. Nude mice treated with saline only were used as the control (Figure 6). The tumors in the control group grew rapidly throughout the treatment. In the group injected with only PFP@IR780@PTX@liposomes without ultrasound irradiation, tumor growth was moderately inhibited, while tumor growth was significantly suppressed after LIFU irradiation. The tumor volume of the PSMA-targeted PFP@IR780@PTX@liposome plus LIFU irradiation group was increased by only 15.03% compared with that of the control group (597.15%), which was significantly lower than that of the PSMA-targeted PFP@IR780@PTX@liposome group, PTX group and PBS+LIFU group (Figure 6B). During the treatment, there was no significant difference in the weight of mice in each group, indicating that PSMA-targeted PFP@IR780@PTX@liposomes had no systemic toxicity in vivo.

**Figure 7:**
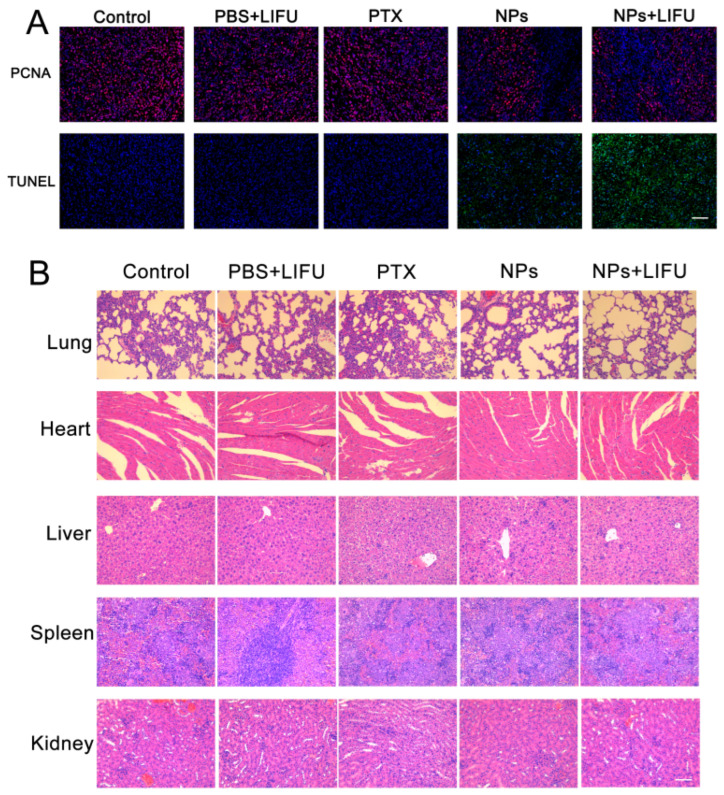
(**A**) Histological assays of tumors with PCNA and TUNEL staining after treatment. (**B**) H&E analysis of the major organ (heart, liver, spleen, lung, kidney) sections after treatments with saline (control) and saline, PTX, PFP@IR780@PTX@liposomes, or PFP@IR780@PTX@liposomes plus LIFU. Scale bar: 50 μm.

In addition, an immunohistochemical method was used to further evaluate the apoptosis of each group. We used terminal deoxynucleotidyl transferase-mediated dUTP-biotin nick end-labeling assay (TUNEL) staining to reveal apoptotic cells (green fluorescence) and proliferating cell nuclear antigen (PCNA) to analyze proliferative cells (red fluorescence). As shown in Figure 7A, the PFP@IR780@PTX@liposome plus LIFU group had the strongest green fluorescence, indicating that the apoptosis rate was the highest. This result suggested that LIFU irradiation could induce the highest antitumor effects. After the treatment was finished, the main organs (lung, heart, liver, spleen, and kidney) of each group were collected, sliced, and stained with hematoxylin and eosin (H&E). Relatively normal histological structures were observed, as shown in Figure 7B. The results showed that sonosensitive phase-changeable nanoparticles caused no significant damage to the different tissues of mice in vivo.

## 3. Discussion

Prostate cancer is one of the most common cancers affecting men. Early detection and treatment are of great importance for prostate cancer. The development of an imaging method and nanotechnology may provide a biosafety and noninvasive way to overcome the limitations of surgery, radiation, and nonspecific chemotherapy in prostate cancer diagnosis and treatment. The nanoprobe mediated image-guided therapy could achieve targeted molecular imaging and the precision therapy of cancers. We produced PSMA-targeted nanoparticles which could recognize prostate cancer specifically. PSMA is a type II transmembrane protein that exists in the cell membrane of prostate epithelial cells, which are the most meaningful target proteins for the diagnosis and treatment of prostate cancers. When the targeted nanoparticles are incubated with PSMA-positive prostate cancer C4-2 cells, the endocytosis efficiency of PSMA-targeted PFP@IR780@PTX@liposomes was significantly higher than that of the untargeted group, indicating that nanoparticles could aggregate into prostate tumor cells actively and efficiently through PSMA-targeting modification to achieve their functions.

Lipid bubbles as well as liposome structures have been investigated as ultrasound contrast agents [16,17,18,19,20] and for imaging-guided therapy and drug delivery. It showed that the lipid shells of bubbles have a good stability for ultrasound imaging in vivo. However, several shortages of bubbles limited their applications, such as large particle size and low drug loading (Table S1). Therefore, the perfluorocarbon lipid nanodroplets have been developed. Perfluorocarbon (PFC) droplets have been investigated for biomedical applications across a wide range of imaging modalities. These PFC nanodroplets can pass through the blood vessels of prostate cancer, enter tumor tissues, and bind to tumor cells, especially aggressive prostate cancer cells, and produce liquid–gas phase transitionstriggered by ultrasound irradiation. Moreover, the nanodroplets have more space for drug loading. Owing to acoustic droplet vaporization (ADV), acoustical microbubbles were produced after ultrasound activation. Our self-made LIFU could deliver ultrasound energy to focal sites, specifically stimulate the phase transition of nanodroplets within the target area, and release the loading drug in a controlled manner. 

In addition, the IR780 and docetaxel were co-loaded in the sonosensitive PFC liquid nanoparticles. IR780 iodide has been used as a light- or ultrasound-responsive agent for cancer SDT, PDT, PTT, and real-time imaging [13,14,15]. It also exhibited preferential accumulation in the mitochondria of tumor cells. Docetaxel, approved by the FDA, has been widelyused in prostate cancer chemotherapyin clinical use [4,5]. Image-guided nanocarrier systems for targeted drug delivery can effectively avoid the toxicity and side effects of docetaxel and improve the efficacy of prostate cancer chemotherapy. Moreover, the fabricated sonosensitive nanoparticles have moderated size and active targeting properties. Therefore, the targeted nanoparticles can pass through the blood vessels of prostate cancer, enter tumor tissues, and bind to tumor cells, especially aggressive prostate cancer cells. Meanwhile, the PFC liquid core could be triggered to produce a gas bubble using ultrasound irradiation owing to acoustic droplet vaporization (ADV). The acoustical microbubbles were accumulated around cancer cells for a long time, benefitting early ultrasound diagnosis and treatment. LIFU with low-intensity ultrasound energy could focus on specific parts and accurately trigger liquid–gas phase transition on targets, preventing potential damage to surrounding tissues and minimizing adverse side effects. Based on acoustic droplet vaporization (ADV) and ultrasound-targeted microbubble destruction (UTMD), the liquid–gas phase transition of liquid fluorocarbon was activated using focused ultrasound, resulting in the controlled release of the encapsulated PTX. Additionally, it increased the permeability of the cell membrane and further enhanced the killing effect of drugs on cells.

Recently, plenty of novel nanoparticles have been developed and used in the diagnosis and treatment of tumors. Meanwhile, with the development of artificial intelligence and machine learning methods, researchers use artificial intelligence technology to diagnose tumors. Scientists from Radboud University Medical Center reported a comprehensive international verification of artificial intelligence (AI) technology for the diagnosis and rating of prostate cancer through research [21]. They found that the AI system can indicate whether the tissue sample contains tumor cells, evaluate the number of tumors in the biopsy tissue, and grade the severity of prostate cancer. It showed that the AI system was comparable to international pathologists. The research results indicated that an AI system may be expected to be introduced into the diagnosis and treatment of prostate cancer as an auxiliary tool, which could help improve the treatment and quality of life of patients with prostate cancer.

In this study, we successfully developed a sonosensitive phase-changeable nanosystem with PSMA-targeted modification for prostate cancer diagnosis and chemotherapy that can specifically identify prostate cancer. The fabricated nanoparticles loaded with PFP and PTX could be used as theranostic agents to detect tumor tissues in real time using ultrasound and PA imaging and achieve targeted and controlled drug release on demand. The intracellular “explosion effect” caused by ADV could enhance the chemotherapeutic effects. It improved the accuracy of treatment and minimized systematic effects, providing an efficient strategy for the real-time imaging and therapy of PCa.

## 4. Materials and Methods

### 4.1. Materials and Reagents

1,2-Distearoyl-sn-glycero-3-phosphoethanolamine-N-[methoxy (polyethylene glycol)-2000 (DSPE-mPEG2000) and 1,2-dipalmitoyl-sn-glycero-3-phospha-tidylcholine (DPPC) were purchased from Xi’an Ruixi Biological Technology (Xi’an, China). IR780, propidium iodide (PI), cholesterol, and docetaxel were purchased from Sigma–Aldrich (Saint Louis, USA).. Anti-PSMA antibodies were purchased from Abcam (Cambridge, UK). Cell Counting Kit-8 (CCK-8) was obtained from Dojindo (Kumamoto, Japan). 4′,6-Diamidino-2-phenylindole (DAPI), 1,1-dioctadecyl-3,3,3,3-tetramethyl indocarbocyanine perchlorate (DiI), and 0.25% trypsin were purchased from Beyotime Biotechnology (Shanghai, China). Chloroform was obtained from Chuandong Chemical Co. Ltd. (Chongqing, China). Perfluoropentane (PFP) was obtained from Apollo Scientific Ltd. (Cheshire, UK).

### 4.2. Synthesis of PFP@IR780@PTX@liposomes

PFP@IR780@PTX@liposomes were fabricated using a facile one-step emulsion method [19,20]. Briefly, DPPC, DSPG, DSPE-PEG2000, cholesterol, and IR780 were dissolved in chloroform at a weight ratio of 10:4:4:2:1. Next, the mixture was subjected to rotary evaporation for 2 h to evaporate the chloroform and form a thin lipid film. After that, 4 mL PBS was added to peel the formed film, assisted by sonication. Then, 4 μL PFP and PTX solution (5 mg/mL) was added to the mixture solution, followed by probe sonication for 5 min at a power density of 125 W. After centrifugation (6000 rpm, 5 min), the precipitates were collected and stored at 4 °C before use. The entire preparation process was performed in an ice bath to avoid PFP liquid vaporization.

### 4.3. Synthesis of PSMA-Targeted PFP@IR780@PTX@liposomes

DSPE-PEG2000 and anti-PSMA polypeptide were dissolved in 4 mL ethanolat 40 °C. EDC was added to react for 15 min, and NHS was added to react for 4 h. The product was passed through a dialysis bag (3500 Da) to remove excess EDC, NHS, and impurity bound peptides and freeze-dried for later use. PSMA-targeted PFP@IR780@PTX@liposomes were fabricated using the same method as above.

### 4.4. Characterization of PFP@IR780@PTX@liposomes

The morphology of PFP@IR780@PTX@liposomes was observed usinga Hitachi 7500 transmission electron microscope (TEM, Tokyo, Japan). Zeta potential and size distribution were measured using Malvern Zatasizer Nanoseries (Malvern, UK). The loading efficiency of IR780 was determined throughlight absorbance at 780 nm. The loading efficiency of PTX was measured by using an HPLC system (Agilent 1200, Santa Clara, CA, USA). The drug loading capacity (LC) was calculated as LC = Drug loaded/Total nanoparticles×100% (n = 3).

### 4.5. Phase Transformation and Ultrasound Imaging of PSMA-Targeted PFP@IR780@PTX@liposomes Triggered by LIFU In Vitro

Phase transformation of PFP@IR780@PTX@liposomes was observed using light microscopy in vitro. PSMA-targeted PFP@IR780@PTX@liposomes were irradiated through LIFU with different acoustic intensities (0.4, 0.8, 1.2 w/cm^2^) for different durations (1, 2, 3 min) in the agarose gel phantom. Then, B-mode and contrast-enhanced ultrasound (CEUS) mode imaging were carried out using ultrasonic equipment (Esaote Mylab 90, Genoa, Italy) with a frequency of 5–12 MHz and a mechanical index (MI) of 0.06. The echo intensity of the region of interest in the ultrasound image was determined using the Ultrasound Image Analyzer (DFY-II type, Institute of Ultrasound Imaging, Chongqing Medical University, China). PBS was irradiated usingLIFU as the control.

### 4.6. Cell Targeting of PFP@IR780@PTX@liposomes In Vitro

The PSMA-positive prostate cancer cell line C4-2 was purchased from the Cell Bank of Shanghai Institute (Chinese Academy of Science, Shanghai, China). Cells were cultured in medium supplemented with 10% fetal bovine serum and 1% penicillin/streptomycin (37 °C and 5% CO_2_). Confocal laser scanning microscopy (CLSM, Nikon, Tokyo, Japan) and flow cytometry (BD Influx, Franklin, USA) were used to assess the intracellular uptake of PFP@IR780@PTX@liposomes. In detail, C4-2 cells in logarithmic growth phase were collected and maintained in CLSM-specified culture dishes for 24 h. Then, the medium in each dish was replaced by serum-free medium containing PSMA-targeted or untargeted PFP@IR780@PTX@liposomes. After 4 h, the cells were washed with PBS and fixed with 4% paraformaldehyde for 15 min immediately. Then, the nucleus was labeled with DAPI for CLSM observation. Flow cytometry was used to analyze the uptake efficiency of the cells. After incubation with PSMA-targeted or untargeted PFP@IR780@PTX@liposome suspension, the cells were digested with 0.25% trypsin and suspended in PBS. Finally, the phagocytosis rate was directly assessed through flow cytometry.

### 4.7. Cellular Apoptosis and Cell Viability Assay In Vitro

To assess the antitumor efficacy in vitro, the C4-2 cells were treated using the following five groups: Control, PTX, PFP@IR780@PTX@liposome, Control+LIFU, PFP@IR780@PTX@liposome+LIFU. A flow cytometer assay was used to evaluate the cellular apoptosis of each group. C4-2 cells were seeded in 6-well plates at a density of 5 × 10^4^ cells/mL and incubated overnight. Then, the cells were treated with 20 μg/mL PTX or 250 μg/mL PFP@IR780@PTX@liposomes for 24 h or 48 h. For the LIFU irradiation groups (Control+LIFU, PFP@IR780@PTX@liposome+LIFU), the cells were irradiated with LIFU (1s on and 1s off for a total of 3 min; 1 MHz; 1.2 w/cm^2^). After incubation for another 24 h, the cells were trypsinized and centrifuged at 1000× *g* for 5 min. Then, the cells were washed three times with ice-cold PBS and resuspended in 200 mL of binding buffer. Thereafter, 5 µL of Annexin V-FITC and 10 µL of PI were added and incubated with the cells for 15 min away from light. The stained cells were analyzed using a flow cytometer. In addition, the viability of C4-2 cells was evaluated using a CCK-8 assay. Briefly, C4-2 cells were seeded in a 96-well plate at a density of 1 × 10^4^ cells/well and allowed to attach for 24 h. After that, for common groups (PTX, PFP@IR780@PTX@liposome), cells were treated with 20 μg/mL PTX or 250 μg/mL PFP@IR780@PTX@liposome for 24 h. For LIFU irradiation groups (Control+LIFU, PFP@IR780@PTX@liposome+LIFU), cells were irradiated by LIFU (1s on and 1s off for a total of 3 min; 1 MHz;1.2 w/cm^2^) after incubation for 1 h. Then, the cells in the LIFU irradiation groups were incubated for another 24 h. After that, 10 μL CCK-8 was added to each well and incubated for 30 min. Then, the plate was subjected to a microplate reader (Thermo Multiskan MK3, Waltham, MA, USA) for cell viability analysis at a wavelength of 450 nm.

### 4.8. In Vivo Phase Transformation and Ultrasound Imaging

BALB/c nude mice (5–6 weeks, 16–18 g) were bred and housed between 19 °C and 22 °C. All the experimental protocols were approved by the Institutional Animal Care and Use Committee of Chongqing Medical University. The experimental operations were carried out in accordance with the protocol approved by the Institutional Animal Care and Use Committee of Chongqing Medical University. The C4-2 xenograft-bearing nude mice were established through a subcutaneous injection of 1 × 10^6^ cells in 100 μL PBS (pH = 7.4) in every mouse. Treatment was initiated when the subcutaneous tumor reached a volume of 80–100 mm^3^. The volume of the tumor was calculated using the following equation: V = length × width^2^/2.

PSMA-targeted PFP@IR780@PTX@liposomes were injected into C4-2-bearing mice through the tail vein at a dose of 200 μL. Two hours later, the tumor was irradiated using LIFU (1 MHz, 1.2 w/cm^2^) at the following conditions: 1 s with a 1 s pause for a total of 3 min. B-mode and CEUS mode imaging of tumors were observed through ultrasonic equipment (Esaote Mylab 90, Italy), and the echo intensity of the tumor region was determined using an Ultrasound Image Analyzer (DFY-II type, Institute of Ultrasound Imaging, Chongqing Medical University, China).

### 4.9. In Vitro and In Vivo Photoacoustic Imaging

To evaluate the photoacoustic performance of PFP@IR780@PTX@liposomes in vitro, various concentrations of nanoparticles were prepared. Photoacoustic imaging of PFP@IR780@PTX@liposomes was observed in the agarose gel phantom at 780 nm using a Vevo LAZR Photoacoustic Imaging System (Visual Sonics Inc., Toronto, ON, Canada). A calibration curve of PA values was obtained using several concentrations of PFP@IR780@PTX@liposomes (0.625, 1.25, 2.5, 5, and 10 mg/mL) in saline solution. The PA performance of PSMA-targeted PFP@IR780@PTX@liposomes was further evaluated in vivo. Tumor-bearing mice were i.v. injected with different PSMA-targeted or untargeted PFP@IR780@PTX@liposome saline solutions (4 mg/mL, 200 µL), and PA images were acquired after injection.

### 4.10. In Vivo Antitumor Therapy

C4-2 xenograft-bearing mice were established as described above. When the tumor volume was approximately 80–100 mm^3^, the mice were randomly divided into five groups (saline, saline+LIFU, PTX, PSMA-targeted PFP@IR780@PTX@liposome, and PSMA-targeted PFP@IR780@PTX@liposome + LIFU), and each group had five mice in each group. Corresponding substances were injected into mice through the tail vein at a dose of 200 μL. After 2 h, tumors were irradiated using LIFU (1 s with a 1 s pause for a total of 3 min; 1 MHz; 1.2 w/cm^2^) for the LIFU irradiation groups (saline+LIFU, PSMA-targeted PFP@IR780@PTX@liposome+LIFU). The tumor volume and body weight of the nude mice were measured and recorded every 2 days. On the 14th day, the nude mice were anesthetized with 5% chloral hydrate, and the tumor as well as the major organ (heart, liver, spleen, lung, kidney) were immediately dissected. Hematoxylin and eosin (H&E) staining was performed for pathological examination. An immunohistochemical examination of the targeted tissue was performed to evaluate the proliferation of these tissues, including a terminal deoxynucleotidyl transferase-mediated dUTP-biotin nick end-labeling assay (TUNEL) and staining for proliferating cell nuclear antigen (PCNA).

### 4.11. Statistical Analysis

All data are presented as the mean ± standard deviation. One-way analysis of variance was utilized for statistical evaluation, and differences were considered statistically significant at *p* < 0.05.

## Data Availability

Not applicable.

## Supplementary Materials

The following supporting information can be downloaded at: https://www.mdpi.com/article/10.3390/ijms24010825/s1.
