# Supplementary material for: Sonosensitive Phase-Changeable Nanoparticle Mediated Enhanced Chemotherapy in Prostate Cancer by Low-Intensity Focused Ultrasound
Source: Int J Mol Sci. 2023 Jan 3;24(1):825. doi: 10.3390/ijms24010825 (PMC9821565; doi:10.3390/ijms24010825)
Supplement: Supplementary file 1 [file ijms-24-00825-s001.zip › ijms-2062536-supplementary.pdf]

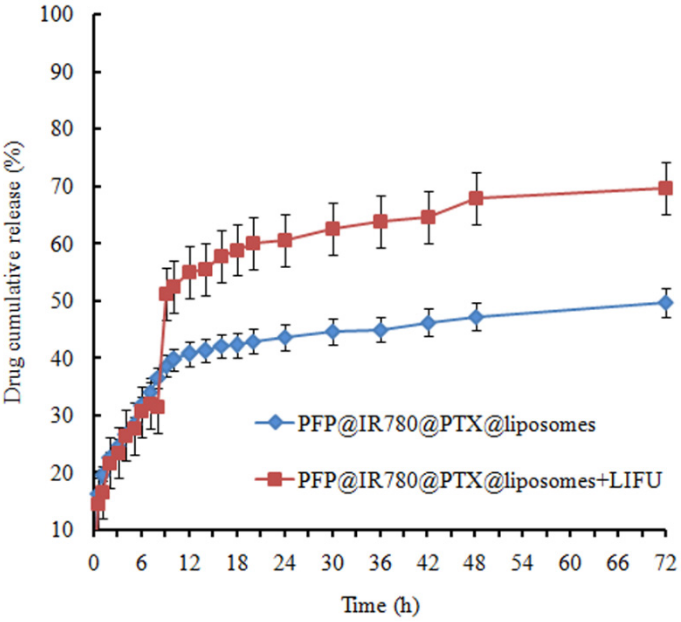

Figure S1. In vitro drug release profiles (LIFU was carried out at 8h)

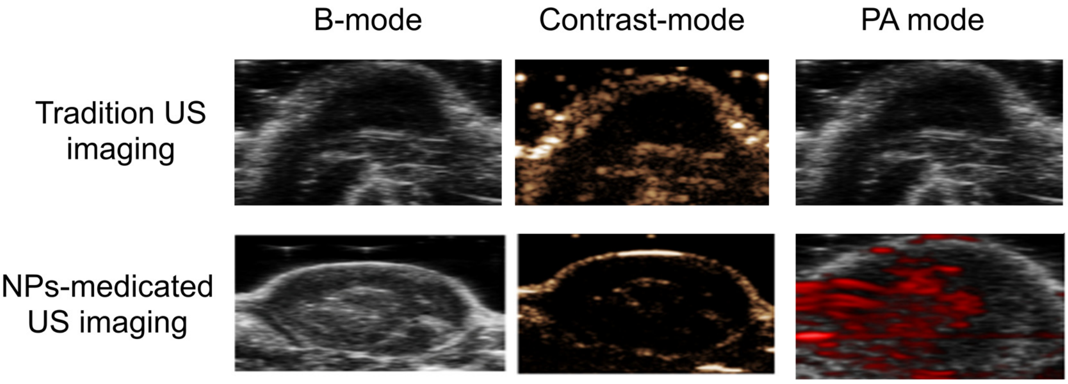

Figure S2. Diagnosis of prostate tumors by traditional US imaging and PFP@IR780@PTX@liposome NPs mediated US/PA imaging in xenograft-bearing nude mice models

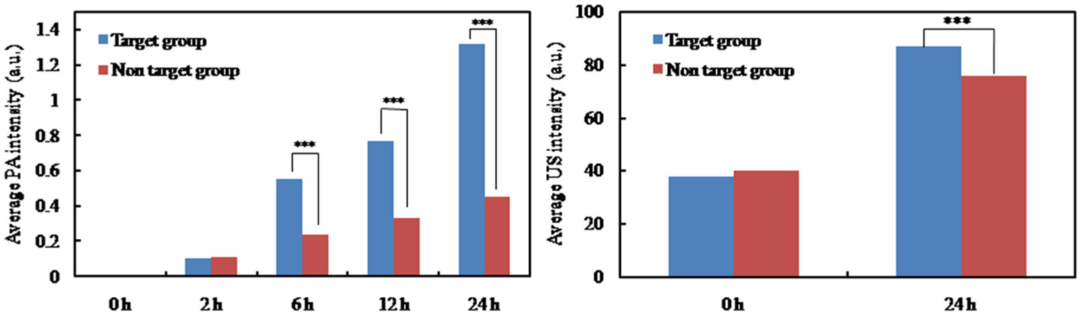

Figure S3. Quantitative analysis of the average PA and US value after PFP@IR780@PTX@liposome NPs injection

Table S1. The application of nanoplatform for cancer theranostic

Platform	Applications	Advantages	Disadvantages	References
Microbubbles	Ultrasound imaging; Drug delivery.	Ultrasound real-time imaging;	Large size; Low loading content; Unstable;	e.g. Ref. 17-18
Nanoparticles	Drug delivery.	Surface targeted modification; Small size; High drug loading.	Not for imaging	e.g. Ref. 16
Nanodroplets	Ultrasound/photoacoustic imaging; Drug delivery.	Surface targeted modification; Small size;	Needs physical stimuli to activate imaging. Medium drug loading.	e.g. The present work
